# The association between sexual function, quality of marital relationship and associated factors in women with a history of ectopic pregnancy: a cross-sectional study in Iran

**DOI:** 10.1186/s12905-023-02635-2

**Published:** 2023-09-21

**Authors:** Fateme Khajoei Nejad, Foozieh Rafati, Shideh Rafati, Neda Dastyar

**Affiliations:** 1 Midwifery Department, Razi Faculty of Nursing and Midwifery, Kerman University of Medical Sciences, Kerman, Iran; 2 School of Nursing and Midwifery, Jiroft University of Medical Sciences, Jiroft, Iran; 3 Social Factors in Health Promotion Research Center, Hormozgan Health Research Institute, Hormozgan University of Medical Sciences, Bandar Abbas, Iran; 4 Student Research Committee, Jiroft University of Medical Sciences, Jiroft, Iran

**Keywords:** Women, Ectopic pregnancy, Sexual function, Marital relationship

## Abstract

**Background:**

Ectopic pregnancy (EP) has many adverse effects on the relationship between couples. The present study aims to assess the association between sexual function (SF), quality of marital relationship (QMR) and associated factors in women with a history of EP.

**Method:**

This cross-sectional study was performed on 220 women with a history of EP in Kerman in 2022. Convenience sampling method was applied. Data were collected using the female sexual function index (FSFI) and the perceived relationship quality components scale (PRQC) questionnaires and were analyzed with descriptive and inferential statistics (median regression) in Stata software version 17. A *P*-value < 0.05 was considered statistically significant.

**Results:**

Of the female participants, 20.4% had sexual dysfunction (SD). Longer duration of marriage (*P* = 0.045) and increase in the number of EPs (*P <* 0.001) were associated with a decrease in SF. A quarter of women experienced poor QMR. Increase in spouse age (*P* = 0.047), longer duration of marriage (*P* = 0.028), and increase in the number of EPs (*P <* 0.001) were associated with a decrease in QMR. There was a significant direct relationship between SF and the QMR (*r* = 0.857; *P <* 0.001).

**Conclusion:**

The present study showed a significant relationship between SF and the QMR in women with a history of EP. Therefore, SF and the QMR are necessary to be considered in future health promotion programs of these women.

## Background

Sexual function (SF) is physiological responses associated with the sexual response cycle that includes desire, excitement, plateau, orgasm, and resolution [1]. SF is characterized by absence of difficulty moving through the stages of sexual response cycle, as well as subjective satisfaction with the frequency and outcome of individual and partnered sexual behaviour [2]. SF is a natural part of human life and a multidimensional concept, which is the result of an interaction between vascular, neurological, and hormonal factors [3]. Women with normal SF experience much less negative emotions and psychological states than women with abnormal SF [4]. SF is influenced by personal (such as demographical, anatomical, hormonal, psychological, and neurological factors, pelvic dysfunction, medications or drug abuse) and interpersonal factors, social norms, cultural and religious values, pregnancy, or pregnancy loss [5–7]. The loss of pregnancy by stillbirth, termination for genetic indications, miscarriage and EP can result in grief, guilt, self-doubt, anxiety and post-traumatic stress disorder [8]. Pregnancy loss is described as a traumatic event for couples, with the potential to change couple’s mental health and lead to disturbance in SF [9, 10].

EP is one of the kinds of early pregnancy loss in the first trimester and a term used to describe any pregnancy which does not implant into the uterine cavity [11]. EP account for approximately 1–2% of all reported pregnancies [12]. This type of pregnancy is often associated with a threat to fertility in the future because of physical damage of fallopian tubes, therefore the loss of fetus along with the increase of susceptibly to infertility expose these women to more anxiety and mental distress [13, 14]. EP is considered one of the most critical issues in women’s reproductive health, which can lead to emotional damage, social problems, financial concerns, and disturbance in the QMR [15]. The QMR is a multidimensional concept and includes various dimensions of marital relationships such as compatibility, satisfaction, happiness, affection, communication, love, attachment, support, integrity, and commitment [16]. The high QMR contributes greatly to a person’s subjective well-being, offers social support, acts as a significant source of happiness, contributes to a better quality of life and enhances a couple’s mental health [17]. In contrast, the low QMR is related to couples and children severe psychological trauma [18]. Unfortunately, 37.9% of women in Malaysia [19], 27% in the USA [20] and 23.5% in Iran [21] complain about the low QMR. The QMR depends on many factors, including the women’s SF and it is mainly regulated by customs, norms and cultural expectations of the society [22]. As a result, it is necessary to conduct more research in the field of SF and QMR in every society and culture [21]; But unfortunately, SF and QMR of different groups of women is a subject that has been neglected to some extent in medical research conducted in the world and especially in Iran [17, 23]. So that, the authors did not found published data on SF and QMR in women with a history of EP. Therefore, this study aimed to fill this information gap by examining the association between SF, QMR and associated factors in women with a history of EP. It is hoped that the results of this study can take a useful step towards improving the physical and mental health of women with EP history around the world.

## Methods

### Participants and study design

This was a descriptive-cross-sectional study. The statistical population of this study included all married women who were in reproductive age and referred to Afzalipur Hospital in Kerman for treatment of EP (based on the patient’s medical record). The samples selected through convenience sampling. Data were collected over a period of 6 months. The inclusion criteria included willingness to participate in the study, age 18 to 40 years with a history of EP in their previous pregnancies, the ability to read and write, and having sexual intercourse in the last 4 weeks. To avoid potential confounding factors, criteria such as a history of neurological and mental diseases, drug use affecting SF (such as antidepressants, antihypertensive drugs, anticonvulsants), having physical diseases affecting SF (such as chronic diseases, hypertension, or diabetes), history of chemotherapy, malignancy, organ transplants, history of women’s surgery (such as unilateral or bilateral salpingectomy, hysterectomy, anterior or posterior colporrhaphy, labiaplasty or perinorrhaphy), History of infertility, experience of unfortunate events (such as the death of loved ones) in the past month, or addiction were considered as exclusion criteria. All inclusion and exclusion criteria were checked using a checklist and according to self-declaration.

To our knowledge, there was no findings of which we could use for sample size calculation. Therefore, we conducted a pilot study with 30 women diagnosed with EP. The standard deviation of the SF score was calculated as 19.95 (*s’* = 19.95). Then, the formula *S* = *s’* ($\frac{\mathbf{n}\mathbf{?}}{\mathbf{n}\mathbf{?}-1})0.5$ was used for calculation of expected standard deviation; with *s’* = 19.95 and *n* = 30 (pilot sample size), *S* = 20.29 (expected standard deviation in the population). Using the formula *n* = $\frac{{{\mathbf{z}}^{2}\mathbf{s}}^{2}}{{\mathbf{d}}^{2}}$, when *z* = 3.84 and *S* = 20.29, the minimum sample size needed to conduct this study was estimated to be 195 subjects. In order to increase the validity of the findings, 240 questionnaires were distributed. Of these, 220 questionnaires were completed and returned. 12 of the identified women refused to participate in the study and 8 women who did not complete all the items of the questionnaire were excluded from the study.

### Data collection instruments

The data collection tool included three questionnaires:

1. Demographic information, which collected information on age of the woman and her husband, occupation of the woman and her husband, education of the woman and her husband, duration of the marriage, number of EPs, mode of delivery, having children, and being related to the husband.
2. FSFI: This tool was developed by Rosen et al. [24] in USA in 2000 to measure sexual functioning in women during the last four weeks before the study, consisting of 19 questions. The sexual functioning questions are related to six parts of sexual desire, arousal, lubrication, orgasm, pain, and satisfaction. The sexual desire section contains two questions. The sexual arousal, and vaginal lubrication sections each have four separate questions. The orgasm, pain, and sexual satisfaction sections have three questions each. The answers to these sections are in the form of a 5-point Likert scale (from “none” = 1 to “always” = 5) or zero, which means no relationship in the last four weeks; the total score of the scale is obtained by adding the scores of the six areas, and a higher score indicates better SF. We consider an FSFI total score of 26.55 as cut score for differentiating women with and without SD [25]. The FSFI in Iran was standardized by Ghassamia et al. (2013) [26] and confirmed in terms of validity and reliability. In the present study, the reliability of this tool was checked by calculating Cronbach’s alpha as 0.75.
3. PRQC: This questionnaire was designed and developed by Fletcher et al. [27] in USA in 2000 and includes 18 questions in the six dimensions of satisfaction, commitment, intimacy, trust, passion, and love (each dimension is measured by three questions). The questions are answered on a 7-point Likert scale (from “not at all” = 1 to “definitely” = 7). The minimum score is 18 and the maximum score is 126, which is obtained from the sum of the scores. Lower scores indicate lower quality and higher scores indicate better QMR in different dimensions. This is also true for each of the six dimensions of the marital relationship that make up the questionnaire. The validity and reliability of this questionnaire in Iran has been confirmed in a study by Sarhadi et al. (2013) [28]. In the present study, the reliability of this tool was confirmed by a Cronbach’s alpha of 0.78.

### Data collection

After obtaining permission from the Ethics Committee of the university and permission from the authorities, the researcher visited Afzalipur Hospital, and after obtaining the approval of the authorities, identified the eligible women using the entry criteria evaluation checklist. The questionnaire was personally completed by the qualified women and then collected. The approximate time to complete the questionnaire was 30 min. Informed written consent was obtained from participants.

### Statistical analysis

Data were analyzed by SPSS software version 20 using descriptive statistics. Continuous variables were reported by mean (standard deviation) and median (interquartile range); categorical variables were reported by number and percent. The normality of the dependent variables (SF score, QMR score) was rejected by the Shapiro-Wilk test (*P <* 0.001). Therefore, median regression was used to estimate relationships between dependent and independent variables by Stata17 software. The QMR score of the women was categorized as weak (if participants scored less than the first quartile), medium (if participants scored between the first and third quartiles), and good (if participants scored more than the third quartile). *P* < 0.05 was considered statistically significant.

## Results

The women were between 18 and 40 years old, and their average age was 27.83 ± 6.76 years. The average age of their spouses was 32.61 ± 6.59 years. The frequency distribution of other demographic variables is shown in Table 1.The median score of SF was 48, and the interquartile range was from 30 to 68. Also, the median score of QMR was 60 with an interquartile range from 41.25 to 83. Based on the cut-off of FSFI, 45 (20.4%) of participants had SD, and based on the categorized quartiles, 55 (25.0%) of women had poor QMR. There was a direct significant correlation between SF and QMR (Spearman correlation = 0.857; *P <* 0.001).

**Table 1:** Frequency distribution of women with a history of EP according to demographic variables

Continuous Variables	Mean (SD^1^)	Median (IQR^2^)	Minimum	Maximum
Woman’s age (year)	27.83 (6.76)	28.00 (22.00–33.00)	18	40
Spouse age (year)	32.61 (6.59)	33.00 (28.00–37.00)	19	54
Duration of marriage	5.76 (4.33)	5.00 (2.00–8.00)	1	19
Number of EPs	1.69 (0.74)	2.00 (1.00–2.00)	1	3
SF score	49.70 (20.86)	48.00 (30.00–68.00)	19	90
QMR score	63.43 (24.99)	60.00 (41.25–83.00)	18	121
**Categorical Variables**	**Frequency**	**Percent (%)**		
QMR score			-	-
Poor Moderate Good	55 109 56	25.00% 49.55% 25.45%		
Women’s occupation	37 183	16.8% 83.2%	-	-
Employed Homemaker				
Spouse occupation	69 101 50	31.4% 45.9% 22.7%	-	-
Laborer Government clerk Jobless				
Women’s education	19 128 73	8.6% 58.2% 33.2%	-	-
Less than high school diploma High school diploma College				
Spouse education	52 89 79	23.6% 40.5% 35.9%	-	-
Less than high school diploma High school diploma College				
Having children	136	61.8%	-	-
Type of delivery	88 48 84	40.0% 21.8% 38.2%	-	-
Vaginal Delivery Caesarean No childbirth				
Number of EPs	105 78 37	47.7% 35.5% 16.8%	-	-
1 2 3				
Consanguineous marriage	136	61.8%	-	-

^1^ standard deviation; ^2^ interquartile range

**Table 2:** Multivariable median regression to find the association between independent variables and SF score in women with a history of EP

Variables	Coefficient	Standard error	*P*-value	95% Confidence interval
Woman’s age (year)	-0.373	0.559	0.506	-1.476, 0.729
Spouse age (year)	0.307	0.390	0.432	-0.462, 1.077
Duration of marriage	-1.507	0.842	0.045	-3.167, -0.153
Number of EPs	-11.832	3.006	< 0.001	-17.760, -5.904
QMR	0.666	0.040	< 0.001	0.587, 0.745
Female occupation (Reference: Employed)				
Homemaker	0.662	4.247	0.876	-7.711, 9.035
Spouse occupation (Reference: Laborer)				
Government clerk	-2.006	3.525	0.570	-8.956, 4.943
Unemployed	-0.558	4.204	0.894	-8.0847, 7.729
Female education (Reference: Less than High school diploma)				
High school diploma	3.728	5.817	0.522	-7.741, 15.197
College	0.913	6.041	0.880	-10.997, 12.824
Spouse education (Reference: Less than diploma)				
High school diploma	3.171	2.102	0.133	-0.974, 7.317
College	6.532	2.173	0.003	2.247, 10.817
Having children (Reference: Yes)				
No	0.168	4.114	0.967	-7.943, 8.279
Type of delivery (Reference: Vaginal delivery)				
Caesarean	-4.830	4.336	0.027	-13.380, -3.719
Consanguineous marriage (Reference: Yes)				
No	-1.657	3.129	0.597	-7.827, 4.512

Based on the findings in Table 2, duration of marriage, number of EPs, and type of delivery affect SF score. By adjusting the effect of other variables, one year increase in duration of marriage, was associated with 1.51 unit decrease in the median of SF score (*P* = 0.045). Each unit of increase in the number of EPs, was associated with approximately 12 unit decrease in the median of SF score (*P <* 0.001). One unit of increase in QMR score, was associated with 0.67 unit increase in the median of SF score (*P <* 0.001). Also, the SF score of women whose husbands had university education was 6.53 higher than women whose husbands had an education lower than high school diploma (*P* = 0.003). Furthermore, the SF score of women who had experienced caesarean was 4.83 less than their counterparts who had had vaginal delivery (*P* = 0.027).

**Table 3:** Multivariable median regression to find the association between independent variables and QMR in women with a history of EP

Variables	Coefficient	Standard error	*P*-value	95% Confidence interval
Women’s age (year)	0.005	0.664	0.994	-1.304- 1.313
Spouse age (year)	-2.330	0.463	0.047	-3.244- -1.250
Duration of marriage	-1.525	0.999	0.028	-3.496- -0.044
Number of EPs	-13.431	3.567	< 0.001	-20.465- -6.397
SF	1.031	0.060	< 0.001	0.911–1.150
Female occupation (Reference: Employed)				
Homemaker	6.671	5.039	0.039	2.264–12.606
Spouse occupation (Reference: Laborer)				
Government clerk	1.742	4.182	0.677	-6.504- 9.989
Unemployed	5.230	4.988	0.296	-4.604- 15.066
Female education (Reference: Less than high school diploma)				
High school diploma	-2.571	6.903	0.710	-16.180- 11.038
College	-3.206	7.168	0.655	-17.339- 10.927
Spouse education (Reference: Less than high school diploma)				
High school diploma	-5.018	4.861	0.303	-14.604- 4.566
College	0.888	5.061	0.861	-9.090- 10.868
Having children (Reference: Yes)				
No	1.032	4.882	0.833	-8.593- 10.657
Type of delivery (Reference: Vaginal Delivery)				
Caesarean	-1.456	5.145	0.777	-11.602- 8.688
Consanguineous marriage (Reference: Yes)				
No	-2.517	3.713	0.499	-9.839- 4.803

Based on the findings in Table 3, spouse age, duration of marriage, number of EPs, and female occupation affect QMR score. By adjusting the effect of other variables, each one year increase in the spouse age, was associated with 2.33 unit decrease in the median of QMR score (*P* = 0.047). One year of increase in duration of marriage, was associated with 1.52 unit decrease in the median of QMR score (*P* = 0.028). Each unit of increase in the number of EPs, was associated with approximately 13 unit decrease in the median of QMR score (*P <* 0.001). One unit of increase in SF score, was associated with 1.03 unit increase in the median of QMR score (*P <* 0.001). Moreover, the median of QMR score of homemakers was 6.67 more than their employed counterparts (*P* = 0.039).

## Discussion

The present study aimed to investigate the association between SF, QMR and associated factors in women with a history of EP in southern Iran. The results showed that 20.4% of female participants had SD. Which seems to be not more than SD reported in other women in Iran [29–31]. The women’s SD reported in other countries is approximately similar to our findings: 25.6% of Chinese women [32] and 28.8% of Brazilian women [33] except women’s SD reported in Polish women (16. 2%) [34] that is lower than our findings. SD in the women with EP history of this study is lower than women with depression [35], breast cancer [36], cervical cancer [37], diabetes [38] and infertility history [39]. However, due to the multifactorial nature of sexual function [35], it is not possible to compare women in different countries with a history of different diseases. Because, many physiological, psychological, medical, social, economic, and cultural factors affect the occurrence of SD [32].

In the present study, quarter of the women had poor QMR. Phillips et al. (2015) [40], in their review, concluded that the QMR of women who had experienced at least one miscarriage was significantly less than women who had never had a miscarriage. It is a fact that the loss of a pregnancy through any event (such as intrauterine fetal demise, miscarriage, or EP) has many psychological complications for the women [13, 41]. These psychological complications can have a destructive effect on the QMR.

The results of this study indicate that there is a direct and significant relationship between the QMR and SF in women with a history of EP. A positive and significant correlation of QMR with the SF has been reported in women with other diseases as systemic sclerosis [42] and breast cancer [29]. Previous studies have also shown that SF is related to marital satisfaction in students [43]. Contrary to these results, Manjula et al. (2021) [44] did not find any relationship between the SF and the QMR in 155 Indian couples. Probably, the difference in the sample size, individual characteristics of the research sample, and the questionnaires used are the cause of difference in the results. It seems that people with better QMR easily share their sexual feelings, needs, and problems, which can help improve their SF.

This study found that longer duration of marriage decrease the score of SF. This finding is consistent with the studies by Kilic et al. (2019) [45], The increase in the duration of marriage is accompanied with changes in other variables such as age, changes in the number of children, and changes in life responsibilities that may affect SF, which were not controlled in this study. Our findings in this case is not consistent with the studies by Madbouly et al. (2021) [46] and Nik-Azin et al. (2013) [47]. As sexual norms, expectations and roles are influenced by cultural and social factors, so this difference may be related to social and cultural characteristics in different societies.

In this study the increased number of EPs was related to decrease SF. Pregnancy loss in a critical situation such as EP that accompanied with increasing mother death risk could lead to anxiety and post-traumatic stress disorder [13] that could probably have adverse effects on women’s SF.

The present study showed that women whose husbands have university education have better SF. Azin et al. (2020) [10] and Darooneh et al. (2015) [48] reached results similar to those of the present study. Men with higher education care more about the satisfaction and SF of their wives.

Women who had a vaginal delivery have higher SF than women who had a caesarean section. This result can be due to the lack of control of factors leading to caesarean section in this study and the increasing statistics of caesarean section, especially elective caesarean section in Iran [49]. This finding is contrary to the studies by Saleh et al. (2019) in Egypt [50] and Kahramanoglu et al. (2017) in Turkey [51]. Most of the studies conducted in this field have shown no significant relationship between SF and type of delivery [52, 53]. It seems that there is a need for more studies to investigate the relationship between the type of delivery and sexual function of women.

The present research showed that the QMR decreased with increase in age and longer duration of marriage. Interpersonal and intrapersonal factors have effect on QMR [16], therefore with the data of the present study, the reason for this relationship cannot be conclusively stated.

Our findings showed that increasing the number of EP increases, decreasing the QMR. This finding may be due to the stress of couples who have experienced EP, which can negatively affect their intimacy and mutual communication and reduce the QMR.

The homemakers in the present study had better QMR than occupied women. Probably, homemakers are away from occupational stress and have more time and energy to establish better QMR.

The limitations of this study were the cross-sectional nature of the survey, collection of self-reported information, which may affect the data reported by women, use of questionnaires, and inability to generalize the results to other societies and cultures. To this end, it is recommended that interventional, case-control, qualitative, and longitudinal studies including simultaneous examination of couples be conducted with larger sample sizes in different society with different cultures.

## Conclusion

The findings of the present study show a significant relationship between SF and the QMR and some demographic characteristics of the studied women, including marriage duration, number of EPs, and type of delivery. Therefore, SF, QMR and associated factors are necessary to be considered in future health promotion programs of these women. Failure to pay attention to the SF of these women can lead to SD, and over time, affect the QMR and family relationships. In addition, many Iranian women do not talk about their sexual and marital problems with health care providers as they consider it a taboo. As a result, it becomes more difficult to identify and treat women who have sexual and marital problems. Considering that women’s sexual health counseling is one of the responsibilities of the treatment team, especially midwives, it is suggested that a women’s sexual health unit be established in health and treatment centers (hospitals) to identify and educate women at risk, provide counseling on sexual issues, and resolve existing problems.

## Data Availability

The datasets used and/or analyzed during the current study are available from the corresponding authors on reasonable request.
